# Development and implementation of a potential coronavirus disease 2019 (COVID-19) vaccine: A systematic review and meta-analysis of vaccine clinical trials

**DOI:** 10.3126/nje.v11i1.36163

**Published:** 2021-03-31

**Authors:** Brijesh Sathian, Mohammad Asim, Indrajit Banerjee, Bedanta Roy, Ana Beatriz Pizarro, Maraeh Angela Mancha, Edwin R. van Teijlingen, Hamed Kord-Varkaneh, Ahammed A Mekkodathil, Supram Hosuru Subramanya, Israel Júnior Borges do Nascimento, Neema Antony, Ritesh G Menezes, Padam Simkhada, Hanadi Al Hamad

**Affiliations:** 1 Rumailah Hospital, Hamad Medical Corporation, Doha, Qatar; 2 Hamad General Hospital, HMC, Doha, Qatar; 3 Sir Seewoosagur Ramgoolam Medical College, Mauritius; 4 Faculty of Medicine, QIUP, Malaysia; 5 Pontificia Universidad Javeriana, Bogotá, Colombia; 6 Bournemouth University, Bournemouth, England, United Kingdom; 7 Faculty of Nutrition and Food Technology, Shahid Beheshti University of Medical Sciences, Tehran, Iran; 8 Manipal College of Medical Sciences, Pokhara, Nepal; 9 University Hospital and School of Medicine, Federal University of Minas Gerais, Belo Horizonte, Minas Gerais, Brazil; 10 Confederation of Epidemiological Associations, India; 11 Imam Abdulrahman Bin Faisal University, Dammam, Saudi Arabia; 12 University of Huddersfield, United Kingdom

**Keywords:** COVID-19, candidate vaccines, Immunogenicity, Solicited and Unsolicited Systemic Adverse Events

## Abstract

**Background:**

To date, there is no comprehensive systematic review and meta-analysis to assess the suitability of COVID-19 vaccines for mass immunization. The current systematic review and meta-analysis was conducted to evaluate the safety and immunogenicity of novel COVID-19 vaccine candidates under clinical trial evaluation and present a contemporary update on the development and implementation of a potential vaccines.

**Methods:**

For this study PubMed, MEDLINE, and Embase electronic databases were used to search for eligible studies on the interface between novel coronavirus and vaccine design until December 31, 2020.

**Results:**

We have included fourteen non-randomized and randomized controlled phase I-III trials. Implementation of a universal vaccination program with proven safety and efficacy through robust clinical evaluation is the long-term goal for preventing COVID-19. The immunization program must be cost-effective for mass production and accessibility. Despite pioneering techniques for the fast-track development of the vaccine in the current global emergency, mass production and availability of an effective COVID-19 vaccine could take some more time.

**Conclusion:**

Our findings suggest a revisiting of the reported solicited and unsolicited systemic adverse events for COVID-19 candidate vaccines. Hence, it is alarming to judiciously expose thousands of participants to COVID-19 candidate vaccines at Phase-3 trials that have adverse events and insufficient evidence on safety and effectiveness that necessitates further justification.

## Introduction

Since December 2019, the world has been experiencing a life-changing pandemic caused by the coronavirus disease 2019 (COVID-19) and its associated etiological agent, i.e., severe acute respiratory syndrome coronavirus-2 (SARS-CoV-2) [1,2]. As of December 27, 2020, this virus had infected more than 79.2 million people and resulted in over 1.7 million fatalities across the globe [3]. The deaths toll resulted from the novel coronavirus infection affected most developed and developing countries worldwide, but it has severely impacted on developing countries, such as Brazil, where vulnerable people are more likely to be associated with coronavirus-related mortality [4]. Therefore, there is an urgent need to prevent COVID-19 infection with the implementation of a safe and effective vaccination program as well as the development of potential therapeutic interventions for treatment.

The primary long-term focus to control the COVID-19 pandemic is implementing a universal SARS-CoV-2 vaccination program that proves safety, efficacy, and cost-effective [5]. To date, there are several potential vaccine candidates in the developmental stage (162 candidate vaccines in preclinical evaluation) (Figure 1a & b) [6]. However, the estimated earliest availability of the possible COVID-19 vaccine is by 2021 [7]. For instance, the Department of Health and Human Services in the United States has initiated the ‘Operation Warp Speed’ which is based on a public-private partnership that is utilizing different pathways to manufacture and deliver a safe and effective vaccine aiming to distribute more than 300 million doses by the beginning of 2021 [8]. Based on the evidence from earlier pandemics, careful planning for the COVID-19 vaccination program is crucial to ensure readiness and accessibility for both public and the healthcare community. To date, only a few comprehensive review articles have evaluated the suitability of candidate vaccines under different phases of evaluation or collated data from ongoing SARS-CoV-2 immunization programs. Therefore, we have conducted the present study to assess the safety and immunogenicity of the novel COVID-19 vaccine candidates and present a contemporary update on the development and implementation of potential vaccine candidates for COVID-19.

## Methodology

The current systematic review followed the Preferred Reporting Items for Systematic Reviews and Meta-Analyses (PRISMA) Statement and Meta-analyses of Observational Studies in Epidemiology (MOOSE) reporting guidelines [9,10]. We prospectively registered the review protocol on Open Science Framework (#osf.io/2jp73/). We have searched (from January 1 to December 31, 2020) MEDLINE (via Ovid platform), PubMed, Embase, Cochrane, clinicaltrials.org and Google for timely reports using different combinations of keywords such as “2019-nCoV”; “SARS-CoV-2”; “Coronavirus Disease 2019”; “COVID 19”; “Vaccine”; “2019 Novel Coronavirus Vaccine”; “2019-nCoV vaccine”; “SARS2 vaccine”; “SARS-CoV-2 vaccine”; “Coronavirus Disease 2019 vaccine” and “COVID 19 vaccine”. Furthermore, we hand-searched the reference list from eligible studies and electronic databases of specific institutional websites and bibliographies (*The Lancet, The Journal of the American Medical Association, and New England Journal of Medicine*) for potentially relevant publications.

### Inclusion/exclusion criteria

We considered studies of any design or any setting which evaluated the safety and security of COVID-19 immunization among non-exposed patients to SARS-CoV-2. We included only full-text available studies published in English. Commentaries, letters to the editor, and review articles were excluded. We have collected the data from eligible studies on the topic of integrative review associated with developing and implementing a new vaccine against coronavirus. We performed an analysis of the most critical issues addressed.

### Outcomes

Outcomes of interest were the presence of side-effects following immunization with COVID-19 vaccination candidates. We defined a medical severe adverse event as any untoward medical contingency that, at any dose, resulted in death, was life-threatening, required hospitalization, or resulted in persistent or significant disability or incapacity. We have mainly analyzed the solicited systemic adverse events (AE). However, some of these studies have recorded unsolicited adverse effects and local site reactions, which were not analyzed due to missing information. Also, serious adverse events were recorded, and the intensity of adverse events was categorized as mild, moderate, severe, and potentially life-threatening. Solicited AE refers to the data collected as part of the uniform collection of information whereas Unsolicited AE refers to information that is volunteered or noted in an unsolicited manner and not as a required data collection element.

### Data extraction

Initially, titles of unique records identified in the systematic search were screened, and, during the full-text evaluation stage, short-listed relevant titles were assessed in detail for suitability of inclusion. Details regarding authors, study setting and period, the origin of studies, sample size, distribution of age and gender, target population, interventions, and outcome measures were extracted. The included articles comprised either single or multi-center studies. Four independent review authors (BS, MA, IB, and ABP) assessed the first and second stage screening, data extraction and risk of bias assessment. Any conflict was resolved by group discussion and the establishment of a consensus.

### Data Analysis and Synthesis

Prevalence was calculated for categorical variables. The decision to select either the fixed-effect or random-effects model relied on the findings of the statistical tests for heterogeneity. The Cochrane Q homogeneity test was utilized to assess the data heterogeneity (significance set at p < 0.10). The fixed-effect model was considered if the studies were found statistically homogeneous. A random-effects model was utilized for studies having statistical heterogeneity. The Higgin’s I^2^ test is the ratio of true heterogeneity to the total variation in observed effects [11]. A rough guide for the interpretation of I^2^ test is as follows: 0-25% (might not be significant); 25-50% (may represent moderate heterogeneity); 50-75% (may represent substantial heterogeneity) and >75% (considerable heterogeneity). Publication bias was visually estimated by analyzing the funnel plots. Pooled estimates were calculated using R 3.5.1 software. Two independent reviewers (ABP and BS) evaluated the methodological quality of the included RCTs using the Cochrane Risk of Bias Tool 2.0 of RevMan version 5.3 (The Cochrane Collaboration, Copenhagen, Denmark) as high, low, or unclear for each item. Eligible studies were assessed on the following items: Bias arising from the randomization process, bias due to deviations from intended interventions, bias due to missing outcome data, bias in measurement of the outcome and bias in selection of the reported results. While non-randomized studies were evaluated through the Risk of Bias in Non-randomized Studies - of Interventions (ROBINS-I) tool [12,13] assessing cofounding, selection of participants, classification of interventios, deviation of intented interventions, missing data, measures of outcomes and selection of reported results.

## Results

**Figure 2:**
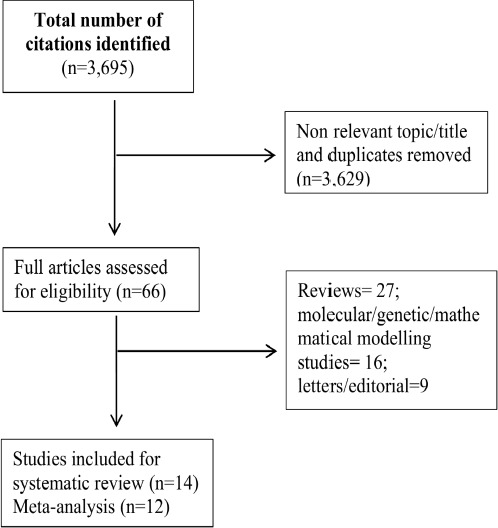
Flow diagram of study selection process for literature search and extraction of data from studies for systematic review

The literature search resulted in a total of 3,695 articles, of which 3,629 were identified as non relevant topic/title and duplicates that were excluded on initial screening. A detailed evaluation of the relevant titles and abstracts based on inclusion and exclusion criteria led to further exclusion of 52 articles from the analysis (Table 1, Figure 2). Finally, fourteen studies that have assessed the safety of COVID-19 vaccination candidates were included in the current systematic review [14-27] and twelve for the meta-analysis [14,15,17-20,22-24,26,27].

### Quality assessment and Publication Bias

The risk of bias was assessed for primary outcomes in eleven included randomized control trials using five domains. Figures (3a-d) show the summary and graph, respectively. The Cochrane Risk of Bias 2.0 tool contains five entries related to bias arising from the randomization process, bias due to deviations from intended interventions, bias due to missing outcome data, bias in measurement of the outcome and bias in selection of the reported results. Three of the studies [25-27] met the criteria for low risk of bias across all domains, under the general heading of randomization to the vaccine administration or placebo, sequence generation 100% percent of the studies were adequately randomized, and at allocation concealment, 60% the studies were considered a low risk of bias and the rest unclear because of unsufficient detail. No studies reported a high risk of bias under blinded assessment of outcome by the administration, minimizing detection bias, but in two studies it was high whether the caregivers and researchers were blinded to the treatment group [17, 24] For missing outcome data, 80% of the studies were at low risk. Under the domain for bias in measurement of the outcome, all studies were considered low risk of bias because they reported an existing protocol, followed, and initially reported the chosen outcomes. For bias in the selection of the reporting results, nine studies were low risk, and the other two remained unclear, and for other biases, eleven studies had low risk because they did not state any important concerns about bias not covered by other domains in the tool. For the three were non-randomized open-label trials we assessed the quality through the ROBINS-I tool, one presented unclear risk on the measures of the outcomes and the rest of the domains on the studies had a low risk on bias under cofounding, selection of participants, classification of intended interventions, missing data and selection of reported results, This reflects the quality and under which rigor vaccine trials are being runned.

### Outcome measures

Among the included 12 studies for meta analysis, 13087 total adverse events were reported from the vaccinated 47019 population. Figure 4a depicts the meta-analysis related to total adverse events among the vaccinated population. The pooled total adverse events was 35%, (95% CI: 26-44%).

### Heterogeneity among included studies

The findings for the heterogeneity test for this meta-analysis to look for the association between the COVID-19 vaccine and total adverse events are displayed towards the bottom of the forest plot in the line. For total adverse events (Q [χ^2^] =768.30, P=0.001, I^2^=99%, tau^2^=0.105 (Figure 4a). As the I^2^ was >25%, a random effect model was considered. Tau^2^ reflects the presence of true heterogeneity among the studies.

### Publication bias and funnel plots

The sensitivity analysis for the above finding demonstrated consistent results. Based on a visual inspection of the funnel plot, there was evidence of publication bias for the included studies (Figure 4b). The funnel plots indicated the presence of studies with large standard error and were not symmetrical.

### Overview of the human trials for COVID-19 vaccine

Table 1-4 shows the characteristics safety, and immunogenicity outcomes of included studies [14-27]. Zhu et al. [14] conducted the first vaccine trial in Wuhan, China, a phase-1, single-center, non-randomized, open-label trial of a recombinant adenovirus type-5 (Ad5) vectored COVID-19 vaccine that uses dose-escalation. This study enrolled healthy adults (18-60 years) who were sequentially given one of the three doses of vaccine, i.e. 5×1010, 1×10¹¹, and 1·5×10¹¹ viral particles assessed for safety, tolerability, and immunogenicity (ClinicalTrials.gov, NCT04313127). The primary outcome measured was the occurrence of adverse events within seven days post-vaccination. The overall safety of the vaccine was also monitored after 28 days of vaccination. The immunogenicity was assessed by measuring the binding antibody responses for the receptor-binding domain and spike glycoprotein, the level of neutralizing antibodies produced in response to the vaccination, and the response of T-cell proliferation. In this study, a higher proportion of subjects in the low and middle-dose group (83% each) and 75% in the high-dose group had at least one adverse reaction within the first seven days post-vaccination. The most frequently observed recurring adverse events for all groups were of mild-to-moderate severity, which mainly included fever (46%), fatigue (44%), headache (39%), and myalgia (17%). The specific (humoral) antibody response against SARS-CoV-2 significantly elevated at day-14 and peaked at 28 days after vaccination, whereas the T-cell response attains the peak on the 14^th^ day after vaccination. The trial’s findings showed promising results in terms of tolerability and immunogenicity of this candidate vector-based COVID-19 vaccine after 28 days of vaccination. A subsequent randomized, double-blinded phase-2 study by Zhu et al. [15] of the same COVID-19 vaccine in 508 participants assessed the safety and immunogenicity. The authors demonstrated that this vaccine is safe and showed marked immunogenicity in most subjects administered with a single dose. Therefore, the candidate COVID-19 vaccine (5 × 10^10^ viral particles) should be tested in a phase 3 effectiveness trial on healthy individuals. Another Phase-1 trial by Jackson et al. [16] enrolled 45 participants to test the RNA-based vaccine’s safety and immunogenicity. Subjects in the higher dose group had higher antibody response, and greater than half the participants were reported to have solicited adverse events. All the participants had developed immunogenicity against SARS-CoV-2. There were no safety issues related to the trial, which support the advancement of this candidate vaccine for testing in a more significant number of participants. Folegatti et al. [17] conducted a single-blind, phase 1/2, randomized controlled trial to assess the safety, reactogenicity, and immunogenicity of the adenovirus-vectored vaccine (ChAdOx1 nCoV-19) and compare it with a meningococcal conjugate vaccine (MenACWY) as control. The candidate vaccine demonstrated considerable safety, humoral, and cellular immune responses and thus can be considered for further largescale evaluation as a phase 3 trial. Xia et al. [18] published an interim analysis of two randomized trials of inactivated vaccine, which reported a lesser frequency of adverse events and acceptable immunogenicity of the candidate vaccine in this ongoing study. A phase 2 single-blind, randomised, controlled, phase 2/3 trial conducted by Ramasamy et al. [19] reported that ChAdOx1 nCoV-19 was better tolerated in older adults than in younger adults and has analogous adverse effects across all age groups after a boost dose. Xia et al. [20] conducted a randomised, double-blind, placebo-controlled, phase 1/2 trial in Henan Province of China on BBIBP-CorV vaccine. The BBIBP-CorV was found safe and well tolerated in all tested doses. Humoral responses were developed 42 days post-immunization.[20] A phase 1, open-label trial published in the *New England Journal of Medicine*, conducted by Anderson et al.[21] on safety and immugenicity of COVID-19 mRNA-1273 vaccine reported that the vaccine produced mild to moderate adverse effects. A randomized, placebo-controlled, phase 1–2 trial conducted by Keech et al. [22] on safety and immunogenicity of the rSARS-CoV-2 vaccine (NVX-CoV2373), 35 days post-immunization with the vaccine was found safe (there was no series adverse effects) and immune response was elicited. In a placebo-controlled, observer-blinded dose-escalation study on 45 healthy volunteers conducted by Mulligan et al. [23] found that BNT162b1 produced dose-dependent local and systemic adverse effects, which were transient and mild to moderate in nature. Walsh et al.[24] conducted a placebo-controlled, observer-blinded, dose-escalation, phase-1 trial on two RNA based vacines (BNT162b1and BNT162b2). In older patients BNT162b2 initiated to produce lesser systemic adverse effects in contrast to BNT162b1 vaccine. [24]. Zhang et al.[25] conducted a randomized, double-blind, placebo-controlled, phase I/II clinical trial in Beijing, China which suggested for efficacy assessment of CoronaVac in future in phase 3 trials at a trivial dose of 3 μg considering the safety, immunogenicity, and production capacity. Polack et al. [26] reported that the RNA vaccine (BNT162b2) was 95% effective against the virus; whereas according to Che et al., [27] adults who obtained the inactivated SARS-CoV-2 vaccine had NAb as well as anti-S/N antibody and experienced few side effects. In addition to these published studies on testing the safety and immunogenicity of COVID-19 candidate vaccines, 162 other ongoing candidate vaccines are currently under clinical evaluation (Phase 1-3).

## Discussion

The systematic review (14 studies) and meta-analysis (12 studies) on COVID-19 vaccines provide the best available evidence that the candidate COVID-19 vaccines are associated with the risk of solicited systemic adverse events and unsolicited systemic adverse events. Our findings suggest that it is essential to account for the adverse events while conducting and implementing the COVID-19 vaccine trials. To date, there is no systematic review on this topic, and most of the earlier reviews are limited in that they have not provided any evidence for adverse events of COVID-19 vaccines. It is important to inform the adverse events of the current COVID-19 pandemic. Previous data from randomized trials are with a smaller sample size comprising different populations and vaccines.

According to the forecasting and fattening of curve theory, there are reduced chances to develop an effective vaccine that can affect the first wave of the COVID-19 pandemic. However, the development of a vaccine in the near future will be useful to prevent subsequent infection waves that may occur later as a seasonal SARS-CoV-2 virus that is supposed to persist in the post-pandemic phase. As COVID-19 is a novel infection, the possible duration of acquired immunity remains unclear, and so is the immunization schedule. Hence, whether single-dose vaccines will confer immunity or require subsequent booster doses can only be ascertained in the future.

### Considerations for a potential COVID-19 vaccine

Phylogenetic analysis of SARS-CoV-2 revealed around 89% nucleotide sequence homology with bat SARS-like coronaviruses (genus Betacoronavirus) identified from China [28]. Therefore, the potential strategies for SARS-CoV-2 vaccine should be guided with the earlier research on vaccine development for other coronaviruses such as SARS (severe acute respiratory syndrome) and MERS (Middle East respiratory syndrome) [29]. Unfortunately, various obstacles are causing the lack of effective vaccine development for the earlier SARS coronaviruses. One of them is the hyperimmune response manifested as eosinophilic infiltration or enhanced infectivity post-immunization with vaccines based on the whole virus or complete spike protein [30]. The mechanism of immunopotentiation is not unusual as a similar response was also reported with a whole virus vaccine for the respiratory syncytial virus (RSV), and these findings are still under investigation [30]. Thus, full safety considerations should be given to avoid any hyperimmune response for a targeted COVID-19 vaccine.

Moreover, research on vaccine development for other coronaviruses does not attract much interest and funding due to relatively lower incidence, and the infection is confined to a specific geographic region [29]. Also, preclinical trials for a candidate vaccine require appropriate funding to conduct studies on small animal models, which was a limiting factor for the previous sporadic outbreaks. Therefore, the development of an effective vaccine for the COVID-19 pandemic is crucial, considering the undesired immunopotentiation, suitability, and availability for healthcare professionals. The availability and suitability for vulnerable populations such as the elderly, pediatrics, or individuals with underlying comorbidities are poignant factors that must be addressed for a potential COVID-19 vaccine [31].

To date, there are three major categories of candidate vaccines, namely the Whole Virus Vaccines (inactive or live-attenuated), Subunit vaccines (recombinant), and nucleic acid vaccines (DNA and RNA) [32]. Out of these available options, vaccines based on nucleic acid have tremendous potential for success and rapid development, seconded by the recombinant-subunit vaccines. Genetic vaccines (DNA or RNA) or viral vector-based vaccines are simple in composition, easy to handle, and readily taken up and translated into protein by host cells, which confer an immunological benefit. Another advantage of RNA vaccines is quick production at a lower cost and has the potential to meet the requirements of mass production in a pandemic. Similarly, DNA vaccine benefits include easy upscaling, low manufacturing cost, high thermal stability, and proven results for human SARS-CoV-1. In addition, it has a better safety profile and enhanced immunogenicity when compared to other types of vaccines [33-35]. Also, it provides useful preclinical and clinical data to be benchmarked for other emerging coronaviruses including, MERS-CoV. RNA vaccine development requires genetic engineering of RNA to attain a strong expression of the viral antigen [33,34].

On the other hand, the neutralizing antibody response to recombinant-subunit vaccines can affect clinical efficacy. However, nucleic acid vaccines are not generally subject to such antibody reactions. Moreover, it eliminates the possibility of an immunodominant response to the desired transgene product. The major drawback is that the vector immunity might negatively influence vaccine effectiveness depending upon vector selection [36]. Also, such vaccines necessitate specific delivery devices to induce desirable immunogenicity.

The classical vaccination strategy for viral infections mainly includes whole virus vaccines, which constitute either live-attenuated or inactive whole virus. The live-attenuated vaccines are preferred as the production process is straight-forward with existing infrastructure and can be benchmarked from various licensed human vaccines. However, the development of infectious attenuated clones of coronavirus for vaccination is a challenging and time-consuming process that depends on the length of the viral genome and requires extensive testing for safety and efficacy. Like live attenuated vaccines, the inactive vaccines can be manufactured using the established infrastructure and production processes available for other licensed human vaccines for SARS-CoV-1, together with adjuvants that can enhance the vaccine’s immunogenicity. Nevertheless, the major challenge is handling the massive amounts of infectious virus, which can be overcome by using an attenuated seed virus. Another difficulty is to confirm the integrity of the antigen or epitope postproduction.

The other type of vaccine candidate for SARS coronaviruses and COVID-19 is the subunit vaccines that trigger an immune response against the spike protein of the virus that prevents its binding with the host’s ACE2 receptor and blocks viral entry. The primary advantage of recombinant protein vaccines is the ease of handling non-infectious particles and the feasibility to use adjuvants that enhance the immunogenicity. However, such vaccines might have limited mass production capacity, and it is challenging to maintain the integrity of the antigen or epitope with high yields.

Worldwide, scientists are working to formulate a robust COVID-19 vaccine rapidly [37]. However, for scientists and physicians, the term “warp speed” should be a matter of concern and scientific research necessitates rigor, discipline, and deliberate caution. Under normal circumstances, the commercialization of any vaccines may take 5-10 years. However, this timeline has been hastened during this pandemic to 12-18 months, including the period for evaluation and confirmation of safety, efficacy, stability, dosage, scalability, and manufacturability. This projection will have to be refined over time, and it is assumed to be one to one and a half years if everything goes smoothly from the point of identifying the vaccine. The process includes small-scale manufacturing for phase I, II, and III clinical trials, followed by regulatory approval and large-scale manufacturing.

During a pandemic, the goal is to compress this timeline for vaccine development and commercialization without impacting safety, which remains critical since the vaccine will be given to a large number of people. Great ideas are not often translated into viable vaccines due to various factors, including reliable scale-up manufacturing or problems during the regulatory approval phase. The manufactured product should be enough to provide the desired dosage and high quality and undergo a robust and consistent process. Currently, there are several vaccine candidates in this race. However, the manufacturing facility requirement varies from type of vaccine production. Therefore, when the winners are announced, the challenge is to secure multiple manufacturing facilities to produce that particular type of vaccine, and there will not be time for alteration.

Notably, any investigational product approved for mass immunization with compromised safety assessment can cause harm. Therefore, it is essential to develop public trust for vaccination trials as volunteers for COVID-19 prevention efforts worldwide. As the quest for an effective SARS-CoV-2 vaccine increases, researchers involved in the developmental process should maintain public trust and not initiate a vaccine trial that either has undermines the standard safety regulations or trials incepted with serious technical loopholes [38]. The safety and efficacy of the COVID-19 vaccine are of the utmost consideration due to the shorter development and testing process. So there might be an underlying suspicion about the effectiveness of vaccines among some segments of the population. For instance, the Phase-3 trial of COVID-19 by AstraZeneca-Oxford University is put on hold after the suspected adverse reaction in study participants from the United Kingdom [39].

Moreover, there are additional challenges and concerns in conducting clinical trials at the time of an ongoing pandemic. It is impossible to predict the location and timing of an infectious outbreak during an epidemic. So it is challenging to select the trial sites that coincide with the availability of the vaccine for testing. Besides, in places where the fatality rate is high, running of randomized controlled trials with a placebo arm may not be feasible due to the higher risk of poor outcomes and ethical aspects. On the other hand, scientific approaches that considers such as aspects as mentioned earlier, may not be speedy to develop vaccines but are scientifically feasible. However, the results can be harder to interpret [40,41]. Recently, the World Health Organization (2019) mentioned that the reluctance or refusal of available vaccines is considered one of the major global health threats and humanitarian crises worldwide [42]. Despite pioneering techniques used for speedy vaccine development in this emergency, it is estimated that the development of an effective vaccine with appropriate safety and efficacy will take at least 12-18 months for mass production and availability [43].

The main strength of this review is associated with its adherence to established methodological features, along with a comprehensive search strategy, public and transparent protocol and meticulous evaluation procedures. Conversely, a significant limitation lies in the differences in study design and heterogeneity between included studies, despite our strict inclusion criteria, probably reflecting the age range, settings, and vaccine types across different studies. Finally, the present comprehensive systematic review provides the best available information on solicited systemic adverse events and unsolicited systemic adverse events of the COVID-19 vaccine. At the same time, contemporary evidence on immunogenicity and efficacy is being generated.

### Expert opinion

The current global pandemic caused by the COVID-19 represents an unprecedented risk, as millions of lives are at stake. The development of a vaccine to circumvent the high death tolls is of paramount importance. This coronavirus virus strain is novel and unique in its genetic makeup, proving foremost pharmaceutical companies a challenge for a definitive answer. The driving force behind the COVID-19 vaccine development is its high infectivity, which affects a vast host of individuals, especially vulnerable populations, i.e. elderly and individuals with concomitant comorbidities. For this reason, the pharmaceutical conglomerates and global leaders in immunology have incepted a unified international clinical trial known as “Solidarity” (ISRCTN83971151). This aims to catapult the synthesis and development of a reliable, safe, and cost-effective vaccine. The current ongoing clinical trials for vaccine development show promising results concerning the candidate DNA and RNA vaccines. Further research and development from this finding will render a potential answer and cure to the current woes and perils experienced by the global healthcare fraternities due to this pandemic.

The real-world implications of this vaccine being a success will be multi-faceted in nature as a vaccine of this design will save countless lives and simultaneously bode positive effects on a proverbial geo-economic disaster caused by stringent lockdown regulations that prohibit trade and business activities. The effect of such a vaccine can therefore be holistically summarized on both a humanitarian and financial basis. The implication of such a vaccine in real-world clinical practice is very likely as the resources for such an endeavor have now been made available due to the extreme need for a vaccine. This juxtaposes abundant resources with a serious lack of time. Unlike the past where vaccine development for previous strains of coronavirus suffered from lack of funding, but ample time. The repercussions of the dearth in knowledge and lack of an effective vaccine for the previous coronaviruses are implicated in the delay of the current vaccine development.

The major limitation in the current scenario is time constraints; thereof, the urgency for such a vaccine to be produced is unnerving and renders a fine line between speed, safety, and efficacy of the vaccine. Notably, a vaccine’s safety must be the prerogative, and no time constraints or external influences should cloud the integrity of the scientific method. A vaccine with inferior safety and efficacy parameters could pose more threats to people’s lives than the actual virus does. There is still an unfathomable amount of exploration and research that needs to be conducted in this field. In the future, the first commercially available vaccine will need further evaluation with post-marketing drug surveillance via phase IV studies. Furthermore, pre-emptive vaccines should be synthesized, considering the different infection waves and genetic variability of the viral strains.

The future of the coronavirus vaccination development is dynamic that needs continuous research on how it can be prevented and circumvented in the future with the use of standard vaccination programs and will eventually require the development of new vaccines for the SARS-CoV-2 virus family on a cyclical basis like that of the influenza virus.

## Conclusion

This systematic review and meta- analysis findings invite a revisit to the reported solicited systemic adverse events and unsolicited systemic adverse events for COVID-19 candidate vaccines. The question in a pandemic is: “Can we justify exposing many people to COVID-19 candidate vaccines at phase-3 trails?”, especially since many such vaccines appear to have adverse events and as yet insufficient evidence on safety and effectiveness. Moreover, further longitudinal studies are needed to better understand the effectiveness of the COVID-19 vaccine for long-term immunogenicity. Currently, there is an urgent need to develop a safe and effective vaccine for COVID-19 and potential therapeutic interventions for treatment. Moreover, the COVID-19 immunization should be accessible to the public as soon as rigorous testing has confirmed the safety and efficacy of the potential vaccine. Upon the development of an effective vaccine, physicians, nurses, and other frontline health care professionals will play a vital role in the mass’ encouragement of COVID-19 vaccination in the community. The vaccination programs should be developed for equitable and judicious use, targeting frontline healthcare professionals and high-risk individuals during the initial phase of vaccine availability, which may be limited. We also need to ensure an equal distribution of vaccines across high- and low-income countries to unsure nobody and no countries stays behind in our fight against COVID-19. It is the responsibility of health regulatory authorities not to endorse a candidate vaccine without sufficient information. The likelihood of achieving public acceptance will depend on scientific evidence based on high-quality trials to endorse the safety and efficacy of the proposed vaccine. The long-term objective of COVID-19 prevention relies on implementing a universal vaccination program with proven safety, efficacy, and cost-effectiveness, which will be socioeconomically beneficial.

## Figures and Tables

**Figure 1a:**
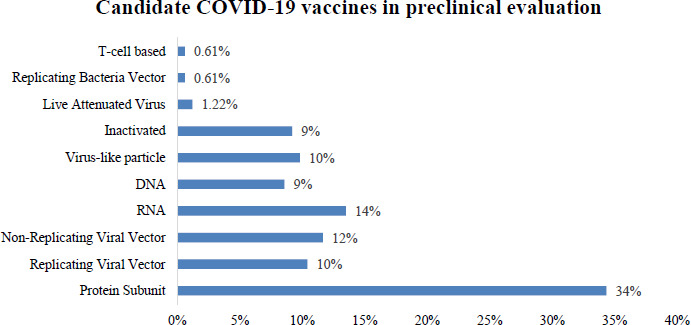
Candidate vaccines in preclinical evaluation (n=162)

**Figure 1b:**
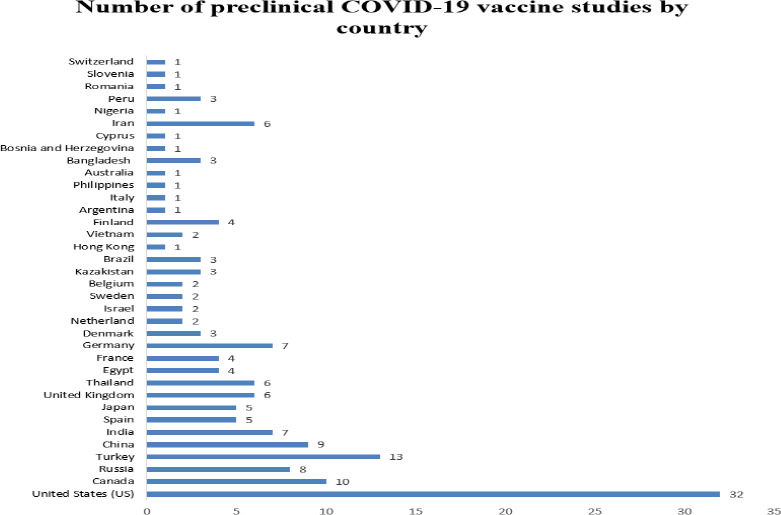
Frequency of preclinical vaccine studies by country (n=162)

**Figure 3a:**
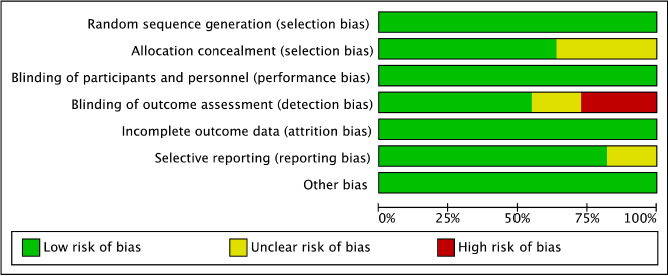
Risk of bias for RCTs

**Figure 3b:**
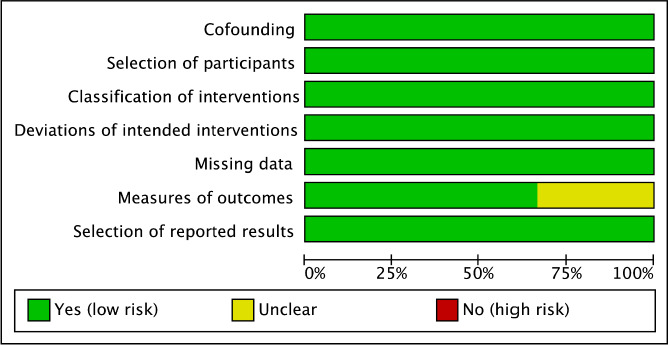
Risk of bias for non-RCTs

**Figure 3c:**
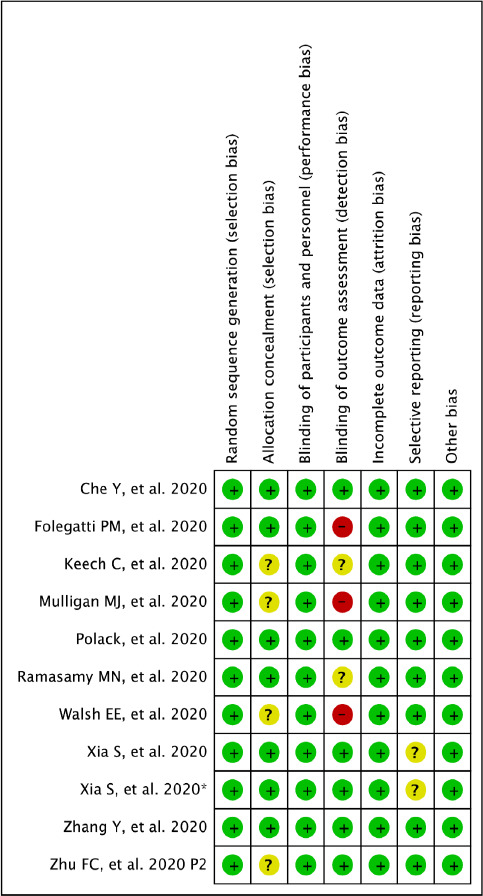
Risk of bias summary for RCTs

**Figure 3d:**
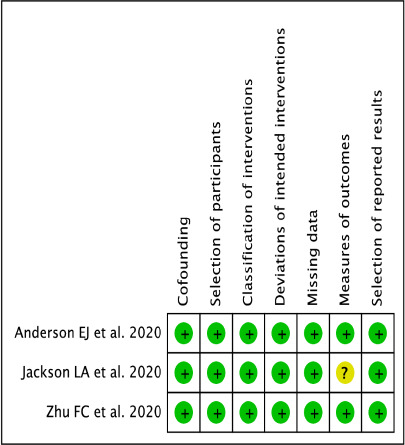
risk of bias summary for non-RCTs

**Figure 4a:**
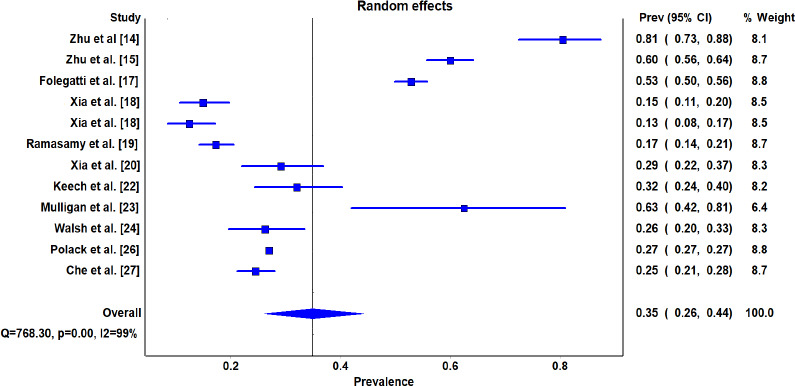
Meta-analysis of the total adverse events in vaccinated population

**Figure 4b:**
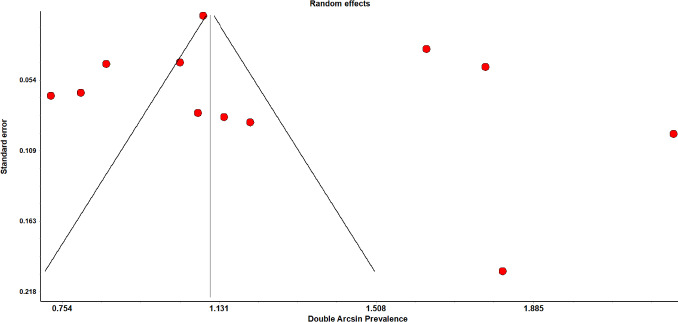
Funnel plot for the total adverse events in vaccinated population

**Table 1:** Characteristics of included studies (n=14)

Study	Years	Trial type	Country	Recruitment dates	Subjects	Sex (M/F)	Age (years)	Target population
**Zhu, et al. [14]**	May-2020	Dose-escalation, single-centre, open-label, non-randomised, phase 1 trial	China	March 16-27, 2020	108	55/53	18-60 (mean 36.3)	Healthy adults [5 × 10^10^ dose (n=36); 1 × 10¹¹ dose (n=36); 1.5 × 10¹¹ dose (n=36)]
**Zhu, et al. [15]**	Jul-2020	Randomized, double-blind, placebo-controlled, phase 2 trial	China	April 11 and 16, 2020	508	254/254	18–83 (39·7±12.5)	Healthy adults [1 × 10¹¹ dose (n=253); 5 × 10^10^ dose (n=129) and Placebo n=126]
**Jackson, et al. [16]**	2020	Phase 1, dose-escalation, open-label trial	US	March 16 and April 14, 2020	45	22/23	18-55 (33.0±8.5)	Healthy adults (n=15 in each group)
**Folegatti, et al. [17]**	2020	Phase 1/2, single-blind, randomised controlled trial	UK	April 23 and May 21, 2020	1077	541/536	18–55 (35, IQR 28–44 years)	Healthy adults (4 groups)
**Xia, et al. [18]**	2020	Randomized, double-blind, placebo-controlled, Phase 1 clinical trial	China	April 12 and May 2, 2020	96	38/58	41.2 ±9.6	Healthy adults (4 groups)
**Xia, et al. [18]**	2020	Randomized, double-blind, placebo-controlled, Phase 2 clinical trial	China	April 12 and May 2, 2020	224	82/142	43.5 ±9.1	Healthy adults (2 groups)
**Ramasamy, et al .[19]**	Nov 2020	single-blind, randomized, controlled, phase 2/3 trial	UK	May 30 and Aug 8, 2020	560	280/280	Gp1:43 years (IQR 33·6–48·0), Gr2: 60 years (57·5–63·0) and Gr3: 73 years (71·0–76·0).	healthy adults aged 18 years and older
**Xia, et al. [20]**	2020	Randomized, double-blind, placebo-controlled, phase 1 trial	China	April 29 and June 28, 2020,	192	90/102	53·7 ±15·6	Healthy adults (18–59 years and ≥60 years)
**Xia, et al. [20]**	2020	Randomized, double-blind, placebo-controlled, phase 2 trial	China	May 18 and July 30, 2020	448	203/245	41·7 ±9·9	Healthy adults aged 18–59 years
**Anderson, et al. [21]**	2020	Phase 1, dose-escalation, open-label clinical trial	United States	April 16 and May 12, 2020	40	19/21	68.7	Healthy older adults stratified according to age (56 to 70 years or ≥71 years) 25 μg or 100 μg
**Keech, et al. [22]**	Sep 2020	Randomized, placebo-controlled, phase 1–2 trial	Australia	May 27 and June 6, 2020	131	66/65	30.8±10.20	Healthy men and nonpregnant women, 18 to 59 years of age
**Mulligan, et al. [23]**	Aug 2020	Phase I/II placebo-controlled, observer-blinded dose-escalation study	United States	May 4 and June 19, 2020	45	51.1%/48.9%	35.4 (range, 19–54 years)	Healthy adults (18–55 years)
**Walsh, et al. [24]**	Oct 2020	Placebo-controlled, observer-blinded, dose-escalation, phase 1 trial	United States	May 4 and June 22, 2020,	195	83/112	18-55 years and (65-85 years)	Healthy adults
**Zhang, et al. [25]**	Nov 2020	Randomized, double-blind, placebo-controlled, phase 1/2 clinical trial	China	April 16 and April 25, 2020	Phase-1 (n=144); Phase-2 (n=600)	165/207	42·6 (9·4)	Healthy adults aged 18–59 years
**Polack, et al. [26]**	Dec 2020	Randomized singleblind control	America, Argentina, Brazil, South Africa, Germany, Turkey	July 27 and November 14, 2020	43548	19,129/18,394	16-55 And >55	Healthy adults above 16 years old
**Che, et al. [27]**	Sep 2020	Randomized double blind control	China	June 2020	742	258/486	41.4 years	Healthy adults aged 18–59 years

**Table 2:** Characteristics of included studies (n=14)-Study outcome, Solicited systemic AEs and Unsolicited AEs

Study	Vaccine	# of doses	Dose schedule (days)	Doses (μg)	Study outcome	Study time point/follow-up	Solicited systemic AEs				Unsolicited adverse reactions			
							Gp 1	Gp 2	Gp 3	Gp 4	Gp 1	Gp 2	Gp 3	Gp 4
**Zhu, et al. [14]**	adenovirus type-5 (Ad5)-vectored	(1:1:2)	0	5 × 10^10^ 1 × 10¹¹, 1.5 × 10¹¹ viral particles	Safety, tolerability, and immunogenicity	Days 7 and 28	30	30	27	-	-	-	-	
**Zhu, et al. [15]**	adenovirus type-5 (Ad5)-vectored	1	0	1 × 10¹¹, 5 × 10^10^ and Placebo	safety and immunogenicity	Days 14 and 28, and month 6	183	96	Placebo	-	19	7	Placebo	
**Jackson, et al. [16]**	mRNA (mRNA-1273)	2	0 and 29	25, 100 and 250	safety and immunogenicity	7 and 14 days after each dose and on days 57, 119, 209, and 394	7	15	14	-	Mild in 69 (21 related to vaccine), Moderate in 19 (12 related to vaccine) and Severe in 2 related to vaccine			
**Folegatti, et al. [17]**	ChAdOx1 nCoV-19 (SARS-CoV-2) vs. MenACWY (Control)	2	0 and 28	5 × 10^10^ viral particles	safety and immunogenicity	Days 3, 7, 14, 28, and 56	42	382	meningococcal conjugate vaccine as comparator	-	12	134	meningococcal conjugate vaccine as comparator	
**Xia, et al. [18]**	Inactivated COVID-19 vaccine (Phase-I)	3	0, 28, and 56	2.5 (low), 5 (medium), and 10-μg (high), control	safety and immunogenicity	Days 28, 90, 180, and 360	5	4	6	-	1	0	4	
**Xia, et al. [18]**	Inactivated COVID-19 vaccine (Phase-II)	2	0 and 14 (Gp-1); 0 and 21 (Gp-2)	5-μg (medium) and control	safety and immunogenicity	Days 28, 90, 180, and 360	5	16		-	2	5		
**Ramasamy, et al. [19]**	ChAdOx1 nCoV-19 (SARS-CoV-2) vs. MenACWY (Control)	2	0 and 28	2·2 × 10^10^ virus particles and 3·5–6·5 × 10^10^ virus	safety and immunogenicity	day 0, 7, 14, and 28	At least one systemic symptom after prime vaccination with the standard dose of ChAdOx1 nCoV-19 by 42 (86%) of 49 participants in the 18–55 years group, 23 (77%) of 30 in the 56–69 group, and 32 (65%) of 49 in the age group of 70 and above.				-			
**Xia, et al. [20]**	BBIBP-CorV	2	0 and 28	2 μg, 4 μg, or 8 μg	safety and immunogenicity	Days 7, 14, 28, 32, and 42	12	11	11	-	-	-	-	-
**Xia, et al. [20]**	BBIBP-CorV	1/2	8 μg on day 0 or on a two-dose schedule of 4 μg on days 0 and 14, 0 and 21, or 0 and 28	safety and immunogenicity	33/84	19/84	15/84	11/84	-	-	-	-		
**Anderson, et al. [21]**	mRNA (mRNA-1273)	2	1 and 29	25 and 100	safety and immunogenicity	Days 1, 15, 29, 36, 43, and 57.	5 (Dose 1); 7 (Dose 2)	5 (Dose 1); 3 (Dose 2)	3 (Dose 1); 8 (Dose 2)	3 (Dose 1); 7 (Dose 2)	3	14		
**Keech, et al. [22]**	full-length wild-type SARS-CoV-2 spike glycoprotein	2	0 and 21	placebo (group A), 25-μg (group B), 5-μg plus Matrix-M1 (group C), 25-μg + Matrix-M1 (group D), single 25-μg + Matrix-M1 + single dose of placebo (group E)	safety and immunogenicity	Days 1, 7, 21, 28 and 35	Dose 1 Gp-A:30%; Gp-B:32%; Gp-C:69.2%; Gp-D:60%; Gp-E:80.7%. Dose 2 Gp-A:19%; Gp-B:24%; Gp-C:92.3%; Gp-D:75%; Gp-E:15.4%				-			
**Mulligan, et al. [23]**	BNT162 mRNA vaccine	2	0 and 21	10 μg, 30 μg or 100 μg	safety, tolerability and immunogenicity	7, 21, 28 and 35 days	25% (3/12 in 10-μg group) to 50% (6/12 each in 30-μg and 100-μg groups) of individuals who received BNT162b1 and by 11.1% (1/9) of placebo group.				-			
**Walsh, et al. [24]**	BNT162b1 and BNT162b2	2	0 and 21	10 μg, 20 μg, 30 μg, and 100 μg	Safety and Immunogenicity	Day 28 and 35	BNT162b1 18–55 years of age 10 μg (3/12) 20 μg (4/12) 30 μg (6/12) 100 μg (6/12) Placebo (1/12) 65–85 years of age 10 μg (3/12) 20 μg (4/12) 30 μg (2/12) Placebo (1/9) BNT162b2 18–55 years of age 10 μg (2/12) 20 μg (4/12) 30 μg (3/12) 100 μg (0/0) Placebo (1/9) 65–85 years of age 10 μg (0/12) 20 μg (1/12) 30 μg (0/12) Placebo (0/9)				-			
**Zhang, et al. [25]**	CoronaVac (an inactivated vaccine candidate)	2	Either day 0 and day 14, or day 0 and day 28	μg and 6 μg and placebo	safety, tolerability and immunogenicity	Day 14, and 28	Phase 1 Dose 1 3 μg group (6/24); 6 μg group (6/24); Placebo group (2/24). Dose 2 3 μg group (1/24); 6 μg group (5/24); Placebo group (1/24). Phase 2 Dose 1 3 μg group (22/120); 6 μg group (21/120); Placebo group (9/60). Dose 2 3 μg group (7/117); 6 μg group (10/118); Placebo group (2/61).				Phase 1 Dose 1 3 μg group (1/24); 6 μg group (2/24); Placebo group (0/24). Dose 2 3 μg group (0/24); 6 μg group (0/24); Placebo group (0/24). Phase 2 Dose 1 3 μg group (22/120); 6 μg group (21/120); Placebo group (9/60). Dose 2 3 μg group (0/117); 6 μg group (0/118); Placebo group (0/61).			
**Polack, et al. [26]**	mRNA	2	0,21	30	Safety and Immunogenicity	1 week, 1 month, 2 months	Systemic events were reported more often by younger vaccine recipients (16 to 55 years of age) than by older vaccine recipients (age 55 +) in the reactogenicity subset and more often after dose 2 than dose 1. Most common reported systemic events were fatigue and headache (59% and 52%, respectively, after the second dose, among younger recipients; 51% and 39% among older recipients)				11678/43252 (27%)			
**Che, et al. [27]**	Inactivated vaccine	2	0,14 or 0,28	100 EU or 150 EU	Safety and Immunogenicity	7 days, 28 days, 12 months	0-14 procedure: 7 days after first and second immunizations, mainly slight fatigue and fever in 10%, 13%, and 14.7% of individuals in the medium-dose, high-dose, and placebo groups, respectively 0-28 procedure: 7 days after the first and second immunizations, mainly including slight fatigue and fever, were reported in 13.3%, 8%, and 9.3% of individuals				Overall adverse reaction rates during the 28 days after immunization were 24%, 27.3%, and 17.3% (0, 14 procedure) and 27.3%, 19.3%, and 12% (0, 28 procedure) in the mediumdose, high-dose, and placebo groups, respectively			

**Table 3:** Percentage of Total Adverse and Serious Adverse Events

Author	Vaccine	Total Adverse events * %(N)	Total Serious Adverse Events ** %(N)
**Zhu, et al. [14]**	adenovirus type-5 (Ad5)-vectored	81 (87/108)	-
**Zhu, et al. [15]**	adenovirus type-5 (Ad5)-vectored	60% (305/508)	6.5 (25/382)
**Jackson, et al. [16]**	mRNA (mRNA-1273)	First dose: 53% (24/45) Second Dose: 80% (36/45)	0 (0/45)
**Folegatti, et al. [17]**	ChAdOx1 nCoV-19 (SARS-CoV-2) vs. MenACWY (Control)	53% (570/1077)	0 (0/1077)
**Xia, et al. [18]**	Inactivated COVID-19 vaccine (Phase-I)	15% (36/240)	0 (0/240)
**Xia, et al. [18]**	Inactivated COVID-19 vaccine (Phase-II)	13% (28/224)	-
**Ramasamy, et al. [19]**	ChAdOx1 nCoV-19 (SARS-CoV-2) vs. MenACWY (Control)	17.3% (97/560)	-
**Xia, et al. [20]**	BBIBP-CorV	29.2(42/144)	0(0/144)
**Xia, et al. [20]**	BBIBP-CorV	-	-
**Anderson, et al. [21]**	mRNA (mRNA-1273)	First Dose: 40% (16/40) Second dose: 63% (25/40) Unsolicited Events irrespective of Dosing: 43%(17/40)	-
**Keech, et al. [22]**	full-length wild-type SARS-CoV-2 spike glycoprotein	32% (42/131)	-
**Mulligan, et al. [23]**	BNT162 mRNA vaccine candidates	63% (15/24)	-
**Walsh, et al. [24]**	BNT162b1 and BNT162b2	26% (41/156)	-
**Zhang, et al. [25]**	CoronaVac (an inactivated vaccine candidate)	Phase 1: Dose 1:31% (15/48) Dose 2: 13% (6/48) Phase 2: Dose 1: 18% (43/240) Dose 2: 7% (17/235)	-
**Polack, et al. [26]**	mRNA	27% (11678/43252)	0.01(4/43252)
**Che, et al. [27]**	Inactivated Vaccine	24.5(146/595)	0(0/595)

*Total Adverse Events = the total number of solicited and unsolicited adverse events from first dose till follow-up

**Total Serious Adverse Events = the total number of solicited and unsolicited serious adverse events from first dose till follow-up

**Table 4:** Immunogenicity outcomes and conclusion of included studies

Study	Seropositivity rates of anti–SARS-CoV-2 IgG ELISA				Neutralizing antibody responses to live SARS-CoV-2				T-cell response post-vaccination				Interpretation
	GP1	Gp2	Gp3	Gp4	GP1	Gp2	Gp3	Gp4	GP1	Gp2	Gp3	Gp4	
**Zhu, et al. [14]**	615·8 (405·4–935·5)	806·0 (528·2–1229·9)	1445·8 (95% CI 935·5–2234·5)	-	14.5 (9·6–21·8)	16·2 (10·4–25·2)	34·0 (95% CI 22·6–50·1)	-	20·8 (95% CI 2·7–34·0)	40·8 (27·6–60·3)	58·0 (39·1–85·9)	-	The Ad5 vectored COVID-19 vaccine is tolerable and immunogenic at 28 days post-vaccination. Humoral responses against SARS-CoV-2 peaked at day 28 post-vaccination in healthy adults, and rapid specific T-cell responses were noted from day 14 post-vaccination.
**Zhu, et al. [15]**	656·5 (575·2–749·2)	571·0 (467·6–697·3)	Placebo	-	19·5 (95% CI 16·8–22·7)	18·3 (14·4–23·3)	Placebo	-	227 (90%, 95% CI 85–93)	113 (88%, 81–92)	Placebo	-	Findings support testing of the Ad5-vectored COVID-19 vaccine at 5 × 10^10^ viral particles in a phase 3 effectiveness trial in healthy adults.
**Jackson, et al. [16]**	2,110 (1,130 – 3,939)	12,130 (8,447 – 17,418)	17,556 (10,869 – 28,358)	-	53.1 (34.0 – 82.9)	120.7 (85.9 – 169.7)	158.0 (130.6 – 191.1)	-	The 25-μg and 100-μg doses elicited CD4 T-cell responses			100-μg dose elicits high neutralization responses and Th1-skewed CD4 T cell responses, coupled with a reactogenicity profile that is more favorable than that of the higher dose.	
**Folegatti, et al. [17]**	157.1 [96.2, 316.9]	210.7 [149.4, 321.6]	1 [1, 1]		87.9 [40, 144.5]	162.9 [61.2, 345.8]	40 [40, 40]		554.3 [311.3, 1017.7]	528.7 [376.3, 603]		61.3 [48, 88]	ChAdOx1 nCoV-19 showed an acceptable safety profile, and homologous boosting increased antibody responses
**Xia, et al. [18]**	415 (288-597)	349 (258-472)	311 (229-422)		316 (95% CI, 218-457)	206 (95%CI, 123-343)	297 (95% CI, 208-424)		The blood lymphocyte subset and cytokine analysis showed no notable changes over time in different groups or substantial differences across groups at a certain time point.			This interim report of the phase 1 and phase 2 trials of an inactivated COVID-19 vaccine showed that patients had a low rate of adverse reactions and demonstrated immunogenicity; the study is ongoing.	
**Xia, et al. [18]**	74 (56-97)	215 (157-296)	-		121 (95-154)	247 (176-345)							
**Ramasamy, et al .[19]**	At both dose levels, and for all dose groups combined, anti-spike IgG responses at day 28 decreased with increasing age. Low-dose groups: 18–55 years, median 6439 arbitrary units [AU]/mL [IQR 4338–10 640], n=49; 56–69 years, 4553 AU/mL [2657–12 462], n=60; ≥70 years, 3565 AU/mL [1507–6345]. Standard dose groups: 18–55 years, median 9807 AU/mL [IQR 5847–17 220], n=43; 56–69 years, 5496 AU/mL [2548–12 061], n=55; ≥70 years, 4156 [2122–12 595]				At day 42 Low-dose groups: 18–55 years, median 161 [IQR 99–233], n=41; 56–69 years, 143 [79–220], n=28; ≥70 years, 150 [103–255]. Standard dose groups: 18–55 years, median 193 [IQR 113–238], n=39; 56–69 years, 144 [119–347], n=20; and ≥70 years, 161 [73–323].				IFN-γ ELISpot responses against SARS-CoV-2 spike protein peaked 14 days after the prime vaccination Standard-dose groups: 18–55 years, median 1187 spot forming cells [SFCs] per million peripheral blood mononuclear cells [PBMCs; IQR 841–2428], n=24; 56–69 years, 797 SFCs [383–1817], n=29; and ≥70 years, 977 SFCs [458–1914].				ChAdOx1 nCoV-19 appears to be better tolerated in older adults than in younger adults and has similar immunogenicity across all age groups after a boost dose.	
**Xia, et al. [20]**	-	-	-	-	22.5 (18.9-26.9)	29.3 (23.8-36.0)	36.7 (29.8-45.2)	-	-				The inactivated SARS-CoV-2 vaccine, BBIBP-CorV, is safe and well tolerated at all tested doses in two age groups. Rapid humoral responses against SARS-CoV-2 were noted from day 4 after first inoculation and 100% seroconversion was found in all participants on day 42.	
**Xia, et al. [20]**	-	-	-	-	14·7 [95% CI 11·6-18·8]	169·5 [132·2-217·1]	218·0 [181·8-261·3])	Placebo: 2·0 [2·0–2·0]	-					
**Anderson, et al. [21]**	323,945 (182,202, 575,958)	1,128,391 (636,087, 2,001,717)	1,183,066 (379,698, 3,686,201)	3,638,522 (1,316,233, 10,058,130)	-	-	530 (337-835)	391 (235-649)	0.089 (-0.025-0.202)	0.035 (0.005-0.065)	0.075 (0.031-0.119)	0.128 (-0.014-0.270)	Adverse events associated with the mRNA-1273 vaccine were mainly mild or moderate. The 100-μg dose induced higher binding- and neutralizing-antibody titers than the 25-μg dose.
**Keech, et al. [22]**	Gp-A: 113.5(93.6-137.6); Gp-B: 575.5 (331.7-998.5); Gp-C: 63160 (47117.3-84666); Gp-D: 47521 (33803.7-66804.6); Gp-E: 2932 (1987.7-4324.8)				Gp-A: 20.0 (20.0-20.0); Gp-B: 41.4 (27.5-62.4); Gp-C: 3906.3 (2555.9-5970.0); Gp-D: 3305 (2205.3-4953.2); Gp-E: 127.6 (81.8-199.1)				Adjuvanted regimens induced antigen-specific polyfunctional CD4+ T-cell responses that were reflected in IFN-γ, IL-2, and TNF-α production on spike protein stimulation.			At 35 days, NVX-CoV2373 appeared to be safe, and it elicited immune responses that exceeded levels in Covid-19 convalescent serum.	
**Mulligan, et al. [23]**	At day 28 10-μg dose: 4,813 U ml–1 30-μg dose: 27,872 U ml–1 100-μg dose: 1,260 U ml–1				14 days after the second dose 180 (10-μg dose level) and 437 (30-μg dose level)				-			These results support further evaluation of this mRNA vaccine candidate.	
**Walsh, et al. [24]**	BNT162b1 18–55 Years of Age (Day 35) 10 μg (5120U/ml) 20 μg (7480U/ml) 30 μg (13940U/ml) 65–85 Years of Age (Day 35) 10 μg (1527U/ml) 20 μg (6399U/ml) 30 μg (4798U/ml) BNT162b2 18–55 Years of Age (Day 35) 10 μg (4717U/ml) 20 μg (7367U/ml) 30 μg (8147U/ml) 65–85 Years of Age (Day 35) 10 μg (3560U/ml) 20 μg (2656U/ml) 30 μg (6014U/ml)				The highest neutralization titers were measured in samples obtained on day 28 (i.e., 7 days after the second dose) or on day 35 (i.e., 14 days after the second dose).				-			BNT162b2 for advancement to a pivotal phase 2–3 safety and efficacy evaluation.	
**Zhang, et al. [25]**	Phase 1 trial 3 μg group (465·8 [288·1–753·1]) versus 24 (100%) in the 6 μg group (1395·9 [955·2–2039·7]) versus two (8%) in the placebo group (89·8 [76·1–105·9]) at 28 days after the second dose in the days 0 and 14 vaccination. Phase 2 trial 3 μg group (GMT 1094·3 [95% CI 936·7–1278·4]) versus 118 (100%) of 118 participants in the 6 μg group (1365·4 [1160·4–1606·7]) versus none of 56 participants in the placebo group (81·0 [79·0–83·0]) at 14 days after the second dose.				Phase 1 trial 3 μg group (5·4 [3·6–8·1] versus 20 (83%) in the 6 μg group (15·2 [11·2–20·7]) versus none in the placebo group (2·0 [2·0–2·0]) at 28 days after the second dose. Phase 2 trial 3 μg group (23·8 [20·5–27·7]) versus 117 (99%) of 118 in the 6 μg group (30·1 [26·1–34·7]) versus none of 60 in the placebo group (2·0 [2·0–2·0]) at 28 days after the second dose in the day 0 and 14 vaccination cohort				Phase 1 trial 3 μg group, 1·2 (0·5 to 1·8) in the 6 μg group, and 1·2 (–0·1 to 2·5) in the placebo group for the days 0 and 28 vaccination cohort.			Taking safety, immunogenicity, and production capacity into account, the 3 μg dose of CoronaVac is the suggested dose for efficacy assessment in future phase 3 trials.	
**Polack, et al. [26]**	BNT162b2 18–55 Years of Age (Day 35) 10 μg (4717U/ml) 20 μg (7367U/ml) 30 μg (8147U/ml) 65–85 Years of Age (Day 35) 10 μg (3560U/ml) 20 μg (2656U/ml) 30 μg (6014U/ml)				-				-			Two 30-μg doses of BNT162b2 elicited high SARS-CoV-2 neutralizing antibody titers and robust antigenspecific CD8+ and Th1-type CD4+ T-cell responses. The 50% neutralizing geometric mean titers elicited by 30 μg of BNT162b2 in older and younger adults exceeded the geometric mean titer measured in a human convalescent serum panel, despite a lower neutralizing response in older adults than in younger adults.	
**Che, et al. [27]**	Seroconversion rates in the medium- and high-dose groups were 89% and 96%, respectively, with GMTs of 23 and 30, respectively, at day 14 after immunization, and 92% and 96% with GMTs of 19 and 21, respectively, at day 28 after immu - nization				approximately 60% seroconversion with GMTs of 387 and 434 at day 14 for the 0, 14 procedure and approximately 50% seroconversion with GMTs of 342 and 380 at day 28				-			Immunogenicity of this vaccine induced a neutralizing antibody response in 95% of the adult population aged 18–59 years, but also that the vaccine had the capacity to elicit anti-N and anti-S antibodies in the ELISA	
